# Gastrointestinal parasites in bullfrogs (*Lithobates catesbeianus*) in aquaculture production units in the Mexican central highlands

**DOI:** 10.1590/S1984-29612023038

**Published:** 2023-06-30

**Authors:** Emmanuel Hernández-Valdivia, Efraín Islas-Ojeda, Rafael Casillas-Peñuelas, Arturo Valdivia-Flores, Alberto García-Munguía

**Affiliations:** 1 Departamento de Ciencias Veterinarias, Universidad Autónoma de Aguascalientes - UAA, Aguascalientes, Mexico; 2 Departmento de Ciencias de los Alimentos, Universidad Autónoma de Aguascalientes - UAA, Aguascalientes, Mexico; 3 Departmento de Ciencias Agronómicas, Universidad Autónoma de Aguascalientes - UAA, Aguascalientes, Mexico

**Keywords:** Aquaculture, bullfrog, frog parasites, Lithobates catesbeianus, parasitology, Aquicultura, rã-touro, parasitos de rã, Lithobates catesbeianus, parasitologia

## Abstract

In Mexico, intensive production of bullfrogs is one of the most important aquaculture activities, due to growing demand for their meat. Frogs can be hosts for several parasites that negatively affect their development and health. The objective of this study was to identify the presence of intestinal parasites in bullfrogs in aquaculture production units. Eighteen bullfrogs aquaculture production units were selected, and 20 animals (n=360) from each farm. Fecal samples were obtained by mucosal scraping and processed using the concentration method. The overall prevalence of intestinal parasites was 70.5%, and all farms had frogs infected by some species of parasite. Two species of parasites were identified: *Eimeria* sp. and *Strongyloides* sp. Significant differences were found regarding parasite prevalence between males and females (73.8% *vs* 58.8%) and regarding tibia length (5.5 *vs* 6.1 cm) and weight (168 *vs* 187 g) between parasitized and non-parasitized frogs. In conclusion, the present study showed a high prevalence of intestinal parasites, and morphometric alterations (weight, snout-cloaca length, radio-ulna length, tibia length and distance between parotid glands) were identified in the parasitized animals. These results provided useful information that will enable establishment of adequate control measures to help minimize the adverse effects of these parasites.

## Resumo

No México, a produção intensiva de rãs-touro é uma das atividades mais relevantes da aquicultura devido à sua crescente demanda. As rãs podem ser hospedeiras de vários parasitos que afetam negativamente seu desenvolvimento e sua saúde animal. O objetivo deste trabalho é identificar a presença de parasitas gastrointestinais (IPs) em rãs de boi em unidades de produção aquícola. Foram selecionadas 18 unidades de produção aquícola de rãs-touro, e de cada unidade 20 animais (n=360). Amostras fecais foram obtidas por raspagem de mucosas e processadas pelo método de concentração. A prevalência geral de IPs foi de 70,5%, todas as fazendas foram infectadas com algum parasita. Duas espécies de parasitas *Eimeria* sp. e *Strongyloides* sp. foram identificadas; encontrando-se diferenças significativas na prevalência entre machos e fêmeas (73,8% *vs* 58,8%), comprimento da tíbia (5,5 *vs* 6,1 cm) e peso (168 *vs* 187 g) entre rãs parasitadas e não parasitadas. Em conclusão, o presente estudo mostrou uma alta prevalência e alterações morfométricas (peso, comprimento do ronco-cloaca, comprimento do rádio-ulna, comprimento da tíbia e distância entre as glândulas parótidas) todas identificadas nos animais parasitados. Esses resultados mostram informações relevantes que permitirão o estabelecimento de medidas de controle adequadas para ajudar a minimizar os efeitos adversos desses parasitas.

## Introduction

The bullfrog is an amphibian species endemic to North America, distributed from southern Canada to the eastern United States and northern Mexico (Ficetola et al., 2007). The first records of aquaculture farms for bullfrogs (*Lithobates catesbeianus*) production in the Americas date back to the late 19^th^ century, on farms with closed ponds and low productive yields (Orchard & Stéfani, 2022). At the end of the 1930s, this species began to be bred outside the United States, with the first BF farm being established in Brazil. In 1925, ranching began formally in Mexico, through an aquaculture production system called "intensive confinement under greenhouse" (México, 2012).

FAO reports that the main frog producing countries are: Taiwan, Brazil, China, and Mexico (FAO, 2018; Orchard & Stéfani, 2022); where Aquaculture Production Units supply 15% of the world market for bullfrogs leg, while the rest is obtained from wild animals (FAO, 2018), these production systems generate annual revenues of more than 40 million dollars with an average production of five million tons of meat per year (Dittrich et al., 2017; Turnipseed et al., 2012). In Mexico, intensive bullfrogs production is one of the aquaculture activities that has taken on greater relevance, due to its growing domestic demand and the export of animals destined for research, teaching and food (México, 2012; Rosado & Arroniz, 2014). In the Mexican central highlands (Aguascalientes, Jalisco and Zacatecas), bullfrog export production in 2015 was 20 tons, while in 2016 and 2017 production increased from 23.5 to 26 tons, respectively; for this reason, aquaculture in Mexico has shown the greatest increase in the primary sector (Islas-Ojeda et al., 2021; SENASICA, 2020).

Frogs can become intermediate or definitive hosts for various species of parasites, which are closely related to their diet, habitat, production system, biosecurity and number of individuals (Pessier & Mendelson, 2017). Intestinal parasites can negatively impact animal development and health (Chero et al., 2014, 2016). However, despite the common occurrence of parasites in amphibians, there are few reports indicating their prevalence and distribution (Lemke et al., 2008).

Bullfrogs aquaculture producers have established diverse strategies for their breeding, establishing mainly intensive production units where many animals are concentrated; where growth, reproduction, temperature, and environment are controlled in order to improve productive parameters (Islas-Ojeda et al., 2021). For this reason, and due to the importance, that ranching is taking on within aquaculture production systems, the objective of this study was to identify the presence of intestinal parasites in bullfrogs in aquaculture production units in the Mexican central highlands.

## Materials and Methods

### Study site

**Figure 1:**
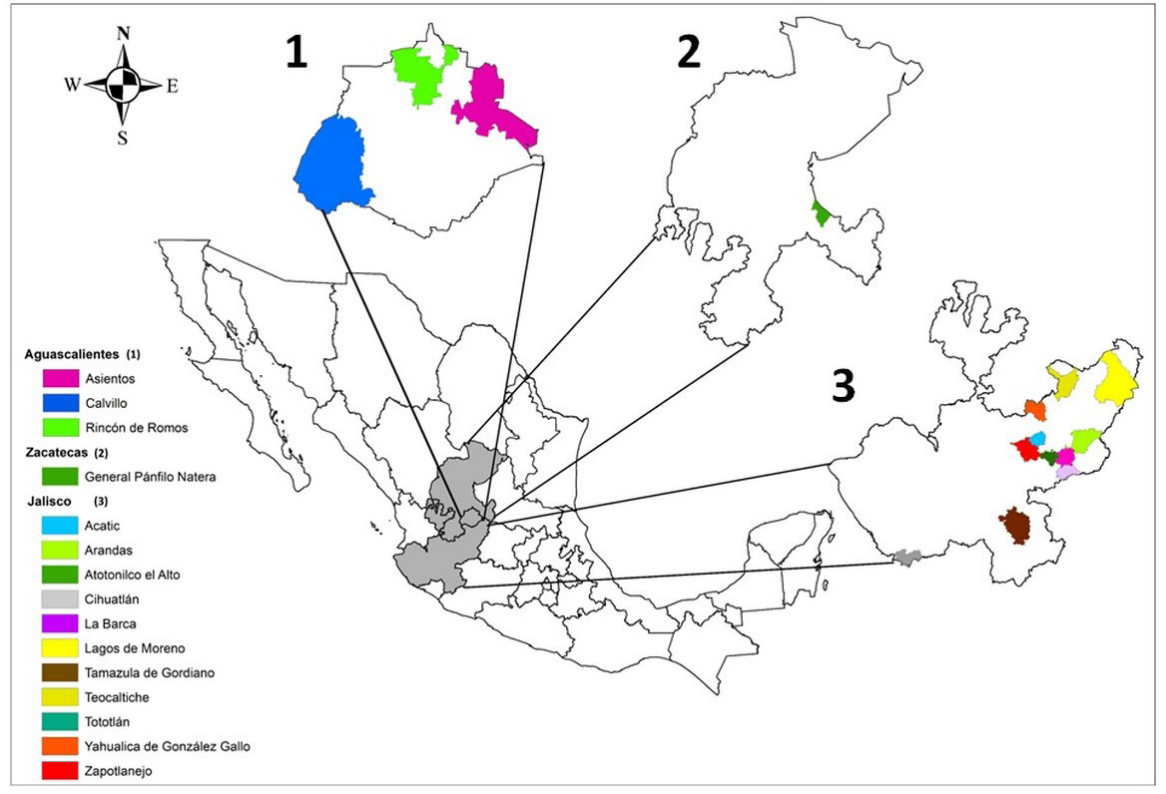
Location of the Mexican central highlands and sampling municipalities where the aquaculture production units are located.

This research was carried out in 18 aquaculture production units for bullfrog meat production in the central Mexican highlands (Aguascalientes, Jalisco, and Zacatecas), where we sought to identify the main intestinal parasites that affect this amphibian species (Figure 1).

The geographical and environmental conditions of each of the states that make up the central Mexican highland’s region are different (Table 1). Due to the location of each of the states, there are slight variations in variables such as: longitude, latitude, temperature (°C), precipitation (mm), relative humidity (%), climate, and altitude (masl).

**Table 1:** Geographical and environmental characteristics of the states that make up the Mexican central highlands.

Item	State		
	Aguascalientes	Jalisco	Zacatecas
Coordinates			
Longitude	102°52'26.40" W, 101°50'06.00" W	105°41'42.00" W, 101°30'39.60" W	104°21'14.40" W, 100°44'31.20" W
Latitude	21°37'20.28" N, 22°27'34.56" N	18°55'33.24" N, 22°45'00.72" N	21°02'30.84" N, 25°07'30.72" N
Temperature (°C)	18.5	20.5	30
Precipitation (mm)	526.8	1 000	510
Relative humidity (%)	59	38.8 - 68.9	72.4
Climate	Semi-dry	Warm sub-humid	Dry and semi-dry
Altitude (masl)	2000	2850	2900

### Animals and sampling

**Figure 2:**
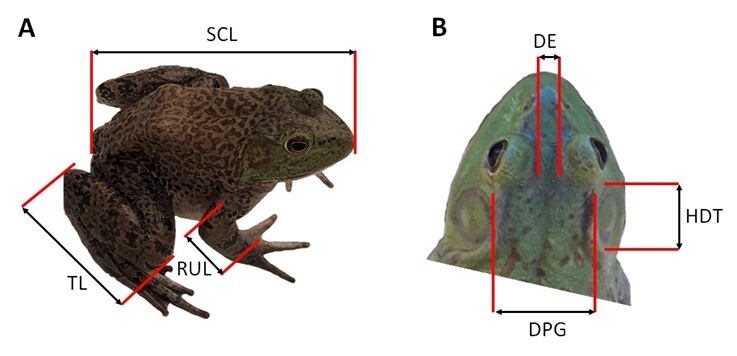
Morphometric measurements on the bullfrogs. (A) SCL: snout-cloaca length; RUL: radio-ulna length; TL: tibia length; (B) DE: distance between the eyes; HDT: horizontal diameter of tympanum; DPG: distance between parotid glands.

A total of 20 animals per aquaculture production units (n = 360) were selected by means of a non-probabilistic randomized design, where the following additional information was obtained for each animal: state, municipality, farm, age and gender. In each of the animals collected, the corresponding morphometric measurements were taken (Figure 2): weight, snout-cloaca length (SCL), radio-ulna length (RUL), tibia length (TL), distance between the eyes (DE), horizontal diameter of tympanum (HDT) and distance between parotid glands (DPG) (Moreno-Rueda et al., 2020; Othman et al., 2022). Subsequently, euthanasia was carried out using the methodology described in the Mexican Official Standard for the Humane Slaughter of Domestic and Wild Animals (México, 1995).

### Parasitological diagnosis

Sample processing was carried out at the Veterinary Parasitology Laboratories of the Center for Agricultural Sciences of the Autonomous University of Aguascalientes. After sacrifice, a complete longitudinal intestinal resection was performed to obtain fecal samples by scraping the mucosa and obtaining 2 grams of feces for subsequent analysis (Rodríguez-Vivas, 2015). Fecal samples were processed using the concentration method with 33% zinc sulfate solution (Bolek et al., 2003; Rodríguez-Vivas, 2015). Identification and quantification of adult helminths, eggs, trophozoites, cysts and oocysts were performed in accordance with established taxonomic keys (Romero, 1994; Anderson et al., 2009; Bolek et al., 2003; Pessier & Mendelson, 2017).

### Data analysis

The data obtained were examined with the Chi-square test (p < 0.05) to detect statistically significant differences. All analyses were performed using statistical software (R, Ver. 3.5.0; Statgraphics, Ver. 16.1.03), considering a confidence level of p < 0.05 as significant.

## Results

The overall prevalence of intestinal parasites in our study was 70.5% (Table 2). Significant differences were observed in the prevalence of intestinal parasites in the aquaculture production units of the states of Aguascalientes, Jalisco and Zacatecas (35.7%, 81.2% and 25.0%, respectively). While in the municipalities, prevalence ranged from 25% (Calvillo, Tototlán and Pánfilo Natera) to 100% (Acatic, La Barca, Lagos de Moreno and Yahualica) were observed, in addition to identifying the presence of intestinal parasites in all the aquaculture production units analyzed. Significant statistical differences (Chi square tests; p < 0.05) were also found with respect to the gender of the animals, with a prevalence of 73.8% in males and 58.8% in females.

**Table 2:** Prevalence of gastrointestinal parasites in bullfrogs according to the different characteristics of the population. (n = 360).

Item	Frogs examined	Positive	Prevalence	*Chi-square test* *(p-value)*
	(No)	(No)	(%)	
Gender				
Male	285	210	73.8^a^	0.02
Female	75	44	58.8^b^	
Stage				
Young	299	210	70.2^a^	0.95
Adult	61	44	72.1^a^	
Farm (No)/City				
**Aguas calientes**	**60**	**23**	**38.3**	0.00
1. Asientos	20	9	45^ab^	
2. Calvillo	20	5	25^a^	
3. Rincon de Romos	20	9	45^ab^	
**Jalisco**	**280**	**226**	**80.7**	
4. Acatic	20	20	100^c^	
5. Arandas	20	9	45^ab^	
6. Atotonilco	20	15	75^abc^	
7. Cihuatlán	20	16	80^bc^	
8. Cihuatlán	20	20	100^c^	
9. La Barca	20	20	100^c^	
10. Lagos de Moreno	20	20	100^c^	
11. Tamazula	20	15	75abc	
12. Teocaltiche	20	10	50^abc^	
13. Teocaltiche	20	20	100^c^	
14. Tototlan	20	5	25^a^	
15. Yahualica	20	20	100^c^	
16. Zapotlanejo	20	16	80^bc^	
17. Zapotlanejo	20	20	100^c^	
**Zacatecas**	**20**	**5**	**25**	
18. Pánfilo Natera	20	5	25^a^	
**General**	**360**	**254**	**70.5**	

^a-c^Means in the same column with different letters are statistically different.

Among the parasites found, the presence of a species of nematode and a protozoan belonging to the genera *Strongyloides* and *Eimeria* was identified, with prevalence of 8.7% and 91.3% respectively in the animals examined (Table 3).

**Table 3:** Prevalence of intestinal parasites and average of eggs and oocysts in feces in bullfrogs (n=360).

Parasite	Prevalence			Abundance Mean (Min -Max)	
	Positive (No)	(%)		Whole specimens	Eggs/cysts (g/f)
*Eimeria* sp.	329	91.3			569 (1-5536)
*Strongyloides* sp.	31	8.7		2.4 (1-9)	113 (1-235)

With respect to morphometrics, significant differences were found in tibia length (5.5 vs. 6.1 cm) and weight (168 vs. 187 g) between parasitized and non-parasitized frogs (Table 4).

**Table 4:** Morphometric differences between parasitized and non-parasitized frogs.

Item	Parasitized	Non-parasitized	SD	*Chi-square test (p-value)*
Morphometry (cm)				
Snout-cloaca length (SCL)	12.4	12.9	2.6	0.45
Radio-ulna length (RUL)	2.4	2.5	0.8	0.62
Tibia length (TL)	5.4	6.1	1.4	0.02
Distance between the eyes (DE)	1.2	1.2	0.6	0.96
Horizontal diameter tympanum (HDT)	1.1	1.1	0.5	0.75
Distance between parotid glands (DPG)	2.6	2.6	0.6	0.94
Weight (gr)				
Young (< 280)	131	161	72.6	0.02
Adult (>280)	317	345	60.2	0.02
Mean	168	187	16.3	0.02

SD: Standard deviation; SCL: snout-cloaca length; RUL: radio-ulna length; TL: tibia length; DE: distance between the eyes; HDT: horizontal diameter of tympanum; DPG: distance between parotid glands.

## Discussion

Infections by intestinal parasites in bullfrogs represent an important animal health problem. which, despite being widely distributed, there are few studies have focused on identification of parasites in aquaculture production units, which could present variations in their prevalence, depending on their geographical location, production systems and biosecurity, generating economic losses due to the negative effect of parasites on animal health (Antonucci et al., 2012). However, even though this species has been introduced into aquaculture production systems in Mexico, there are no published studies on the parasites that are infecting this amphibian species in the wild or in production systems in our country (Cabrera-Guzmán et al., 2021).

Our study observed an overall prevalence of intestinal parasites in aquaculture production units of 70.5%, with a high prevalence in APU in the state of Jalisco (81.2%) compared to those identified in Aguascalientes and Zacatecas (35.7% and 25.0%, respectively). These results are similar to those reported in other studies, where a prevalence of 81.3% was identified in wild animals in Argentina and from 43 to 46% in the United States (Lemke et al., 2008; Miller et al., 2009; González et al., 2014); in production animals a prevalence of 1.7% is reported in aquaculture production units from Brazil (Antonucci et al., 2012). These results suggest that wild frogs suffer a higher rate of infection by gastrointestinal parasites while production animals have a lower degree of parasitosis. However, there are few studies that indicate the behavior of parasitosis in farm animals.

Worldwide there are few studies oriented on determining the prevalence of intestinal parasites in amphibians. being mainly studied wild frogs obtained directly from their habitat, where several authors report the presence of gastrointestinal parasites in 100% of the animals examined (Cabral et al., 2011; Chen et al., 2021; Li et al., 2013; Sou et al., 2018). Being identified mainly parasites belonging to trematodes, acanthocephalans and cestodes, where the prevalence reported for each of the parasites found is variable due to the different environmental conditions, present in each study area (Bolek et al., 2003; Bolek & Janovy, 2007; Chen et al., 2021; Li et al., 2013; Sou et al., 2018). These results indicate that the overall prevalence of intestinal parasites found in our study is high and similar to the observed by other authors.

In the present study, significantly higher prevalence was identified in males (73.8%) than in females (58.8%). This was similar to what had been reported by other authors in different species of anurans, where higher presence of parasites was identified in males compared to females (Chero et al., 2016), mainly attributed to hormonal differences between males and females, being testosterone responsible for influencing the parasite load in these animals (Chero et al., 2014).

In our study, two different types of parasites were identified. The most frequent was *Eimeria* sp., with a prevalence of 91.3% (329/360), few studies carried out in wild anurans report a prevalence of 12.0% of infected animals (27/224) with this type of coccidia, probably infected by ingestion of oocysts eliminated through the feces of previously infected animals (Bolek et al., 2003); while there are no reports of the presence of these parasites in farm animals. These results suggest that infections in production animals would be the result of the biosecurity conditions established in each of the aquaculture production units and the frequent replacement of the water in which infected and healthy animals live. Another parasite found was a nematode belonging to the genus *Strongyloides* sp*.*, with a prevalence of 8.7% in the animals observed (31/360), although this nematode is not one of the most common in frogs, it has been reported parasitizing the intestine of several species of frogs and amphibians in America (Miller et al., 2009; Pessier & Mendelson, 2017). Our results shed light on the presence of two different species of parasites infecting bullfrogs, which have been reported in few studies compared to other species of cestodes, trematodes and acanthocephalans frequently reported in frogs (Cabrera-Guzmán et al., 2021; Chen et al., 2021; Kuzmin et al., 2020; Sou et al., 2018).

In the present study, significant differences (Chi square tests; p < 0.05), in morphometric values were identified between parasitized and non-parasitized frogs, with differences in the length of the tibia (5.4 vs. 6.1 cm) and the average weight of the animals (168 vs. 187 g), where parasitized adult frogs had a weight of 317 g, compared to healthy animals with a weight of 345 g. Similar studies conducted in different species of anurans show a decrease in the average weight of parasitized compared to healthy animals. Likewise, they report a decrease in morphometric measurements of animals with the presence of gastrointestinal parasites (Chero et al., 2016). This negative effect has been previously reported in relation in different animal species affected by *Eimeria*, where damage to intestinal cells prevents adequate nutrient absorption and decreases weight gain and growth, as well as allowing the presence of secondary infections (Alcala-Canto et al., 2020; Bangoura et al., 2014; Salinas et al., 2019). Our results demonstrate that intestinal parasitosis can significantly affect weight gain in affected animals, which in frogs decreases the size and weight of muscle masses located in the legs, which are considered the main production target of intensive aquaculture production units for bullfrogs meat production and one of the main products marketed worldwide (FAO, 2009; Hatziioannou & Karatsivou, 2020; Ribeiro & Toledo, 2022).

For this reason, knowing the prevalence of gastrointestinal parasites in frogs will allow establishing prophylactic measures for prevention and control of this type of parasites, as well as providing pertinent information for veterinarians and producers that will allow them to establish adequate measures for the integral control of parasites and avoid parasitosis and its adverse effects.

## Conclusion

In conclusion, the present study showed a high prevalence of gastrointestinal parasites in aquaculture production units of bullfrogs, especially in males and in farms in different municipalities of the state of Jalisco. Two species of parasites reported for the first time in aquaculture production units in Mexico (*Eimeria* sp*.* and *Strongyloides* sp*.*) were identified. These intestinal parasites can have a negative impact on the animals due to their capacity to generate intestinal damage and alter nutrient absorption. Morphometric alterations were identified in the parasitized animals, which presented lower weight and decreased length of the tibia. The results reported in our study provide relevant information that will allow the establishment of adequate control measures to help minimize the adverse effects of these parasites, which are mainly related to decreased in growth, poor feed conversion and a decreased in meat production, which could cause great economic losses to aquaculture producers. It also opens the possibilities for various parasitological studies to better explain the dynamics of the existing parasite populations.
